# The Multivariate Regression Statistics Strategy to Investigate Content-Effect Correlation of Multiple Components in Traditional Chinese Medicine Based on a Partial Least Squares Method

**DOI:** 10.3390/molecules23030545

**Published:** 2018-03-01

**Authors:** Ying Peng, Su-ning Li, Xuexue Pei, Kun Hao

**Affiliations:** 1 State Key Laboratory of Natural Medicines, Key Lab of Drug Metabolism and Pharmacokinetics, China Pharmaceutical University, 24 Tong Jia Xiang, Nanjing, Jiangsu, 210009, China; 1020162518@cpu.edu.cn (Y.P.); cpu_pxx@126.com (X.P.); 2 China National Center for biotechnology Development, Beijing 100039, China; lisn@cncbd.org.cn

**Keywords:** *panax ginseng* saponins, partial least squares regression, fingerprints

## Abstract

Amultivariate regression statisticstrategy was developed to clarify multi-components content-effect correlation ofpanaxginseng saponins extract and predict the pharmacological effect by components content. In example 1, firstly, we compared pharmacological effects between *panax ginseng* saponins extract and individual saponin combinations. Secondly, we examined the anti-platelet aggregation effect in seven different saponin combinations of ginsenoside Rb1, Rg1, Rh, Rd, Ra3 and notoginsenoside R1. Finally, the correlation between anti-platelet aggregation and the content of multiple components was analyzed by a partial least squares algorithm. In example 2, firstly, 18 common peaks were identified in ten different batches of *panax ginseng* saponins extracts from different origins. Then, we investigated the anti-myocardial ischemia reperfusion injury effects of the ten different *panax ginseng* saponins extracts. Finally, the correlation between the fingerprints and the cardioprotective effects was analyzed by a partial least squares algorithm. Both in example 1 and 2, the relationship between the components content and pharmacological effect was modeled well by the partial least squares regression equations. Importantly, the predicted effect curve was close to the observed data of dot marked on the partial least squares regression model. This study has given evidences that themulti-component content is a promising information for predicting the pharmacological effects of traditional Chinese medicine.

## 1. Introduction

Traditional Chinese medicine is becoming more and more popular all over the world because of their safety and efficacy [1,2,3,4]. A thorough understanding of the chemical composition of traditional Chinese medicine is essential for a comprehensive assessment. The chemical constituents and their amounts in herb are different, due to growing conditions, such as climate, soil, the drying process, the harvest season, et al. The studies of traditional Chinese medicines mainly focus on the chemical content, pharmacological, and pharmacokinetic studies of the reported main ingredients [5,6,7,8]. However, the relationship between multiple components of traditional Chinese medicine and the efficacy of herbal medicines should be a focus of attention, which strongly restricts dose-effect application, especially in model research. Therefore, the herbal medicine analysis and the curative effect should be associated with each other, which can guarantee the clinical safety and effectiveness of traditional Chinese medicine. The traditional dose-effect models, such as direct model, effect compartment model, and indirect response model, were not appropriate to be applied in traditional Chinese medicine. Therefore, we apply a statistical strategy to select a partial least squares model for predicting the connection of the pharmacological effect with the content of multiple components in the traditional Chinese medicine. A partial least squares model was firstly used in the chemical field, and the wavelength of the infrared reflection spectrum was regarded as the independent variable to predict the chemical composition [9]. With the development of the partial least squares method, it has been used in more and more fields, such as chemistry, engineering, economics, et al. [10,11,12]. When compared with the ordinary least squares regression model, the partial least squares method has some outstanding advantages in solving problems, such as a small number of observations and multiple correlations. Since the partial least squares method is based on the singular value decomposition method of multidimensional array, the key advantage of using PLSR is that the multiple input variables are permissible [13]. Additionally, it is also possible to use a hybrid input of multiple types of variables such as saponin combinations, identified fingerprint and component content.

Panaxginseng saponins extract is one of the most frequently used traditional Chinese medicines due to its tonic functions for more than 2000 years in Asia. It showed wide range of pharmacological effects in cancer, diabetes, cardiovascular system, immune system, and central nervous system [14,15,16,17,18,19,20,21,22]. In the present study, *panax ginseng* saponins extract was taken as example to investigate the correlation between its protective effects on the cardiovascular system and its content of multiple components based on a partial least squares model. As a novel attempt, this was an effective complement to the traditional dose-effect model. The multivariate regression statistic strategy could assess the contribution of each component to the efficacy of herbal medicine, and, in turn, predict the potential efficacy of herbal medicine according to its components compositions. This would have important application value in the research and development of traditional Chinese medicine.

## 2. Materials and Methods

### 2.1. Chemicals and Reagents

Notoginsenoside R1 (NGR_1_), Ginsenoside Rb1 (GRb_1_), Ginsenoside Rd (GRd), and Ginsenoside Ra3 (GRa_3_) were provided by Yunnan Plant Pharmacy Co. Ltd. (Kunming, China). Ginsenoside Rg_1_ (GRg_1_) and Ginsenoside Re (GRe) were provided by the Chinese National Institute for the Control of Pharmaceutical and Biological Products (Beijing, China). The *panax ginseng* saponins extract was purchased from Zelang Chemicals Co. Ltd. (Nanjing, China). The *panax ginseng* saponins extract manufactured by Zelang Group was a yellowy powder made from the *panax ginseng* root, which were washed, microwave-dried, and smashed into powder. *Panax Ginseng* root contains ginsenosides, polysaccharides, organic acids, alkaloids, esters, enzymes, trace elements, and other nutrients.

Diphosphate Adenosine Disodium (ADP-2Na), heparin, and sodium azide were purchased from Sigma-Aldrich Co. Ltd. (Shanghai, China). Sodium chloride, potassium chloride, sodium bicarbonate, magnesium chloride, sodium dihydrogen phosphate, and calcium chloride were purchased from Nanjing Chemical Reagent Co. Ltd. (Nanjing, China). d-glucose was provided by Chinese Pharmaceutical Group Shanghai Chemical Reagent Co. Ltd. (Shanghai, China). Acetonitrile and phosphoric acid of HPLC grade were purchased from Merck Co. Inc. (Shanghai, China). All of the the reagents were of analytical grade.

### 2.2. Animals

*Male* SD rats (200–250 g) were purchased from Beijing Vital River Laboratory Animal Technology Co., Ltd. (Beijing, China). Animals were kept on a 12 h light/dark cycle at the animal center of China Pharmaceutical University for a minimum of three days before experiments. All of the experiments were approved by the animal ethics committee at China Pharmaceutical University.

### 2.3. Cell Cultureand Hypoxia

The H9C2 embryonic rat heart-derived cells were obtained from American Type Culture Collection (ATCC; VA, USA) and maintained in Dulbecco’s modified Eagle’s medium supplemented with 10% *v*/*v* fetal bovine serum and 100 mg/mL penicillin/streptomycin at 37 °C in a humidified atmosphere containing 5% CO_2_. The cells were fed every 2–3 days, and sub-cultured once they reached 70–80% confluence. To induce hypoxia, cells were placed in a Gas Pak EZ Gasgenerating Pouch System for 8 h and were incubated with serum free and glucose free DMEM, as described previously. Asnormoxia control, serum free DMEM was added to cells and incubated for 8 h in normoxia condition (21% O_2_).

### 2.4. Instruments

QX-100 platelet aggregation analyzer was obtained from Shanghai Fudan University Instrument factory (Shanghai, China). Drug analysis was performed with a Shimadzu HPLC-UV system (Tokyo, Japan), comprising a LC-10AD pump, an UV-visible detector, and a CTO-10A column oven. Milli-Q pure water instruments were from Millipore Co. Ltd. (Hong Kong, China).

### 2.5. Assay of Individual Saponin Combinations (Example 1)

#### 2.5.1. The Preparation of the *Panax ginseng* Saponins Extract

The plant part used in our study was the dried roots of *panax ginseng.* The ginseng that we used was provided by the ginseng herbs GAP production base of Guizhou Yi Bai Pharmaceutical Co., Ltd. (Fusong County, Jilin Province, China), which was identified as the dried roots of *panax ginseng* C.A. Mey. (Fam. Araliaceae) by Professor Sun Qishe in Shenyang Pharmaceutical University (China). 400 g of *panax ginseng* was pulverized, mixed, and steeped in 2400 mL of drinking water for 0.5 h at room temperature before a 1 h sonication-enhanced extraction. The extract was separated by filtration, and the residue was re-extracted with 1600 mL of water. The pooled extract was concentrated under reduced pressure at 40 °C and was modified with hydroxypropyl methylcellulose at 0.3% (grams per milliliter) before addition of water to 800 mL to yield *panax ginseng* saponins extract. The *panax ginseng* saponins extract was stored at 15 °C pending use. Dissolve and mix in pure water before use. The contents of GRb_1_, GRg_1_, GRd, NGR_1_, GRe, and GRa_3_ were 34.98%, 26.36%, 8.76%, 7.93%, 4.61% and 3.82% from HPLC result, respectively (Table 1). The total contents of the six saponins reached 86% of *panax ginseng* saponins extract, suggesting that this six saponins were the main material basis for *panax ginseng* saponins extract. 

#### 2.5.2. The Preparation of Plasma Dilutions

NaCl 4 g, KCl 0.1 g, d-glucose 0.5 g, NaHCO_3_ 0.75 g, MgCl_2_·6H_2_O 0.2135 g, NaH_2_PO_4_ 0.0325 g, heparin 6.67 mg (1000 U), and CaCl_2_ 0.10 g were dissolved in pure water, and then 0.5 mL 0.1% NaN_3_ solution were added. Transfer the solution to a 500 mL volumetric flask and then add pure water to the scale. 

#### 2.5.3. The Preparation of Phosphate Buffer Solutions

Na_2_HPO_4_·12 H_2_O 71.644 g was dissolved in 1000 mL pure water to get 0.2 M Na_2_HPO_4_ buffer. NaH_2_PO_4_·H_2_O 27.610 g was dissolved in 1000 mL pure water to get 0.2 M NaH_2_PO_4_ buffer. 72 mL Na_2_HPO_4_ buffer (0.2 M) and 28 mL NaH_2_PO_4_ buffer (0.2 M) were mixed well to get phosphate buffer solutions (0.2 M) for pending use.

#### 2.5.4. The Preparation of ADP-2Na

Add 4.27 mg ADP-2Na into a 5 mL volumetric flask and add phosphate buffer solutions (0.2 M) to the scale. Take 100 µL ADP-2Na solutions into a 5 mL volumetric flask and add phosphate buffer solutions (0.2 M) to the scale before use.

#### 2.5.5. Determination of Anti-Platelet Aggregation

QX-100 platelet aggregation analyzer should be turned on 1 h before the test. Put the diluted plasma tubes with stir beads into preheating hole of the analyzer. After 5 min, move the tube into the test hole of the analyzer. Put the electrode into the tube and press stirring key, and then 5 µL diluted ADP-2Na solution was added into the plasma sample. The initiation of aggregation occurred after 15 to 30 s, and then recorded the platelet aggregation resistance value (Ω).

The inhibition of platelet aggregation in vitro was calculated by formulas as follows: Aggregation inhibition rate (%) = (platelet aggregation resistance value in control group-platelet aggregation resistance value in test group)/(platelet aggregation resistance value in control group). The inhibition of platelet aggregation in vivo was calculated by formulas, as follows: Aggregation inhibition rate (%) = (platelet aggregation resistance value before administration-platelet aggregation resistance value after administration)/(platelet aggregation resistance value before administration).

#### 2.5.6. The Comparison of Anti-Platelet Aggregation between the *Panax ginseng* Saponins Extract and Individual Saponin Combinations

Individual GRb_1_, GRg_1_, GRd, NGR_1_, GRe, and GRa_3_ weighed accurately were mixed and dissolved in pure water to obtain individual saponin combinations. The vitro study was performed in seven groups as follows: saline group, *panax ginseng* saponins extract at 50 µg/mL, *panax ginseng* saponins extract at 100 µg/mL, *panax ginseng* saponins extract at 200 µg/mL, individual saponin combinations at low dose group (the each individual saponin dose was same to the individual saponin dose in *panax ginseng* saponins extract at 50 µg/mL), individual saponin combination at middle dose group (the each individual saponin dose was same to the individual saponin dose in *panax ginseng* saponins extract at 100 µg/mL), and individual saponin combination at high dose group (the each individual saponin dose was same to the individual saponin dose in *panax ginseng* saponins extract at 200 µg/mL). The *panax ginseng* saponins extract and the individual saponins combinations were added into 0.45 mL blank plasma with heparin 20 U/mL, respectively. Determine the platelet aggregation resistance value and calculate the anti-platelet aggregation rate.

In vivo study, forty-two male rats were randomly assigned to seven groups to receive an intravenous *panax ginseng* saponins extract at saline group, 5 mg/kg, 10 mg/kg, 20 mg/kg, individual saponin combination at low dose group (the each individual saponin dose was same to the individual saponin dose in *panax ginseng* saponins extract at 5 mg/kg), individual saponin combination at middle dose group (the each individual saponin dose was same to the individual saponin dose in *panax ginseng* saponins extract at 10 mg/kg), and individual saponin combination at high dose group(the each individual saponin dose was same to the individual saponin dose in *panax ginseng* saponins extract at 20 mg/kg). The plasma samples were collected at predose and 3 h after drug administrations. Determine the platelet aggregation resistance value and then calculate the anti-platelet aggregation.

#### 2.5.7. The Anti-Platelet Aggregation of Different Individual Saponin Combinations 

Different individual saponin combinations were prepared by weighing different saponins accurately into pure water. We selected six major saponins (GRb_1_, GRg_1_, GRd, NGR_1_, GRe, and GRa_3_) in the *panax ginseng* saponins extract to set seven different individual saponin combinations based on the uniform design. Then, the anti-platelet aggregation effect of seven different combinations was studied in vitro (Table 2) and in vivo (Table 3). In vitro, the seven saponin combinations were added into 0.45 mL blank plasma from male SD rats with heparin 20 U/mL, respectively. Determine the platelet aggregation resistance value and calculate the anti-platelet aggregation rate. In vivo, forty-two male rats were randomly assigned to seven different individual saponin combinations to receive an intravenous administration. The plasma samples were collected at predose and 3 h after drug administrations. Determine the platelet aggregation resistance value and calculate the anti-platelet aggregation rate. In order to analyze the correlation between the anti-platelet aggregation effect and individual saponin by partial least squares regression, we changed both the total concentrations and the individual saponin concentration in seven different combinations toobserve the differences of the anti-platelet aggregation effect in different groups. For six saponins, each saponin included seven concentrations (level 1–7), both in vitro and in vivo studies, according to uniform design of *U_7_(7^6^)*.

#### 2.5.8. The Partial Least Squares Method between Different Individual Saponin Combinations and Anti-Platelet Aggregation Effect

The content of six saponins in seven different individual saponin combinations were six independent variables. The anti-platelet aggregation effect was the only dependent variable. The correlation between the content of six saponins and the anti-platelet aggregation effect was analyzed by partial least squares regression. The content-effect data matrix was constructed with the observation in columns and the responses as variables in rows. The content of six saponins in any saponin combination were the X-matrix and the anti-platelet aggregation effect was the Y-matrix. Data processing was carried out by SIMCA-P 11 software (Umetrics, Umeå, Sweden). Because there was only one dependent variable, the partial least squares regression one prediction model was selected. Partial least squares regression analysis was employed to process the acquired content-effect data matrix following established methodology [23,24,25,26].

### 2.6. Assay of Fingerprint (Example 2)

#### 2.6.1. The Fingerprint Studies of *Panax ginseng* Saponins Extract from Different Origins

The HPLC fingerprints of ten different batches of *panax ginseng* saponins extract (S1–S10) were obtained using the established method [27] with slightly modification. An Agilent ZORBAX SB-C_18_ (250 mm × 4.6 mm, 5 μm) column was used with acetonitrile (A) and 0.05% phosphoric acid (B) in gradient elution mode. The elution profile was 0–7 min (5% A), 7–10 min (5%–10% A), 10–30 min (10%–30% A), 30–40 min (30%–35% A), 40–45 min (35%–50% A), 45–60 min (50%–70% A), 60–90 min (70%–90%A); the detection wavelengths were 203 nm; the column temperature was set at 25 °C, and the flow rate was 1.0 mL/min. The prepared *panax ginseng* saponins extract solutions from ten different origins were filtrated through 0.45 μm filter before HPLC-UV analysis. The peak areas of 18 identified components (P1–P18) were adopted as independent component variables.

#### 2.6.2. Cell Treatment

Cell studies were divided into 12 groups: sham control group, ischemia reperfusion (IR) group, IR group combined with ten different batch of *panax ginseng* saponins extract (S1–S10) at 300 µg/mL. In the sham control group, cells were maintained in Dulbecco’s modified Eagle’s medium supplemented with 10% *v*/*v* fetal bovine serum. IR model group was established through hypoxia 3 h/oxygen 2 h. In *panax ginseng* test group, the cells were treated with *panax ginseng* saponins extract (300 µg/mL) for 2 h before induction of hypoxia 3 h/oxygen 2 h.

#### 2.6.3. MTT Assay

MTT assay was performed to determine the effect of *panax ginseng* saponins extract on the cell viability of the H9C2 embryonic rat heart-derived cells. Firstly, cells (5 × 10^4^ cells/mL) were seeded in 96-wells. After two days of culture, different batches of *panax ginseng* saponins extract (S1–S10) was added and incubated for 24 h. Then, the MTT solution at a final concentration of 0.5 mg/mL was added to each well while continued to incubate for 4 h at 37 °C. At the end of the incubation, the culture medium was discarded and 150 mL DMSO was added to each well to dissolve dark blue formazan crystals. The absorbance was read at 570 nm using FLUO star Omega plate reader (BMG LABTECH, Ortenberg, Germany).

#### 2.6.4. Animal Studies

Rats were divided randomly into 12 groups: sham control group, ischemia reperfusion (IR) group, IR group combined with ten different batch of *panax ginseng* saponins extract (S1–S10) at 100 mg/kg/d, with 10 rats in each group. In sham and IR group, rats were given saline by oral gavage for 15 days before surgical operation. In *panax ginseng* test groups, rats were administered with *panax ginseng* saponins extract at 100 mg/kg/d by oral gavage for 15 days before IR operation. Myocardial ischemia-reperfusion (IR) rat model was performed using the method described by Luo et al [28] with slight modifications. Briefly, Sprague-Dawleyrats were anaesthetized by intraperitoneal injection of 10% chloralhydrate (350 mg/kg), and maintained by bolus injection of 10% chloral hydrate (60–80 mg/kg, i.v.) during anaesthesiaas required. The neck was dissected and a tracheostomy was performed to provide artificial ventilation (60 strokes/min at a tidal volume of 10 mL/kg). The fourth and fifth ribs on the left side of the chest were cut to perform the thoracotomy and to incise the pericardium. The hearts were gently exteriorized and a 5/0 silk suture and were passed around the left anterior descending coronary artery. The suture then was ligated and the ends of this ligature were passed through a small vinyl tube to form a snare. After 30 min of ischemia, the snare was removed gently and myocardium was reperfused for 90 min. Ischemia was confirmed by ST-segmentelevation in the electrocardiogram and color changes in the ischemia myocardial area. Rats in the sham group underwent thoracotomy but the left anterior descending was not ligated. After the IR surgery, the animals were sacrificed with an overdose of 10% chloral hydrate (500 mg/kg, i.v.), blood samples were collected from the abdominal aorta. After standing for 30 min of the blood sample, the serum was separated by centrifugation at 4 °C (3000 r/min, 10 min), and stored in −80 °C for further study.

#### 2.6.5. Determination of cTnI, CK and LDH

cTnI, CK, and LDH were three cardiac injury markers. cTnI was one of the three subunits of troponin, which is a protein that was unique in the myocardial. CK referred to creatine kinase, LDH referred to lactate dehydrogenase, these two indicators were often increased in myocardial injury or necrosis. The levels of cTnI were analyzed using an ELISA kit (USCN Life Science, Inc., Wuhan, China), and the data were measured on a microplate reader (Bio-Tek Instruments, Inc., Winooski, VT, USA). The levels of CK and LDH were performed using a suite of commercial kits (Jian Cheng Bioengineering Institute, Nanjing, China). For cell study in vitro, culture medium was collected to measure cTnI, CK, and LDH by the kits mentioned above, according to the manufacturer’s instructions. For animal study in vivo, the serum levels of cTnI, CK, and LDH were analyzed.

#### 2.6.6. The Relevance Analysis between Fingerprint and Drug Effects

The peak areas of 18 identified components (P1–P18) during the fingerprint study of ten batches of *panax ginseng* saponins extract (S1–S10) were regarded as 18 independent component variables. For each component, the corresponding peak area was divided by the average area of the reference peak in ten *panax ginseng* saponins extract samples for nondimensionalization (Table 4). The values of cTnI, CK, LDH and anti-platelet aggregation rate were the four dependent variables. The correlations between the relative content of eighteen independent components and the four dependent variables were analyzed by partial least squares regression. The relative content-effects data matrix was constructed with the observations in columns and the responses as variables in rows. The relative contents of the eighteen components from ten different origins were the X-matrix and the four effectsfrom ten different origins were the Y-matrix. Data processing was carried out by SIMCA-P 11 software.

## 3. Results

### 3.1. Results of Individual Saponins Combination (Example 1)

#### 3.1.1. The Comparison of Anti-Platelet Aggregation between *Panax ginseng* Saponins Extract and Individual Saponins Combination

During in vitro study of part 2.5.6, the anti-platelet aggregation effect of individual saponin combination could reach 90.3%, 92.9%, and 93.0% than that of *panax ginseng* saponins extract at 50 µg/mL, 100 µg/mL, and 200 µg/mL, respectively. Similarly, during in vivo study of part 2.5.6, the anti-platelet aggregation effect of individual saponins combination could reach 89.2%, 91.5% and 92.3% than that of *panax ginseng* saponins extract at 5 mg/kg, 10 mg/kg and 20 mg/kg, respectively. Thus, the six saponinsofNGR_1_, GRg_1_, GRd, GRe, GRb_1_, and GRa_3_ for saponin combination could be recognized as the pharmacological markers of *panax ginseng* saponins extract both in vitro and in vivo. These results shown that the individual saponin content of NGR_1_, GRg_1_, GRd, GRe, GRb_1_, and GRa_3_ in *panax ginseng* saponins extract could be datasets as independent variables in partial least squares method.

#### 3.1.2. The Anti-Platelet Aggregation Effect of Different Individual Saponin Combinations

**Figure 1:**
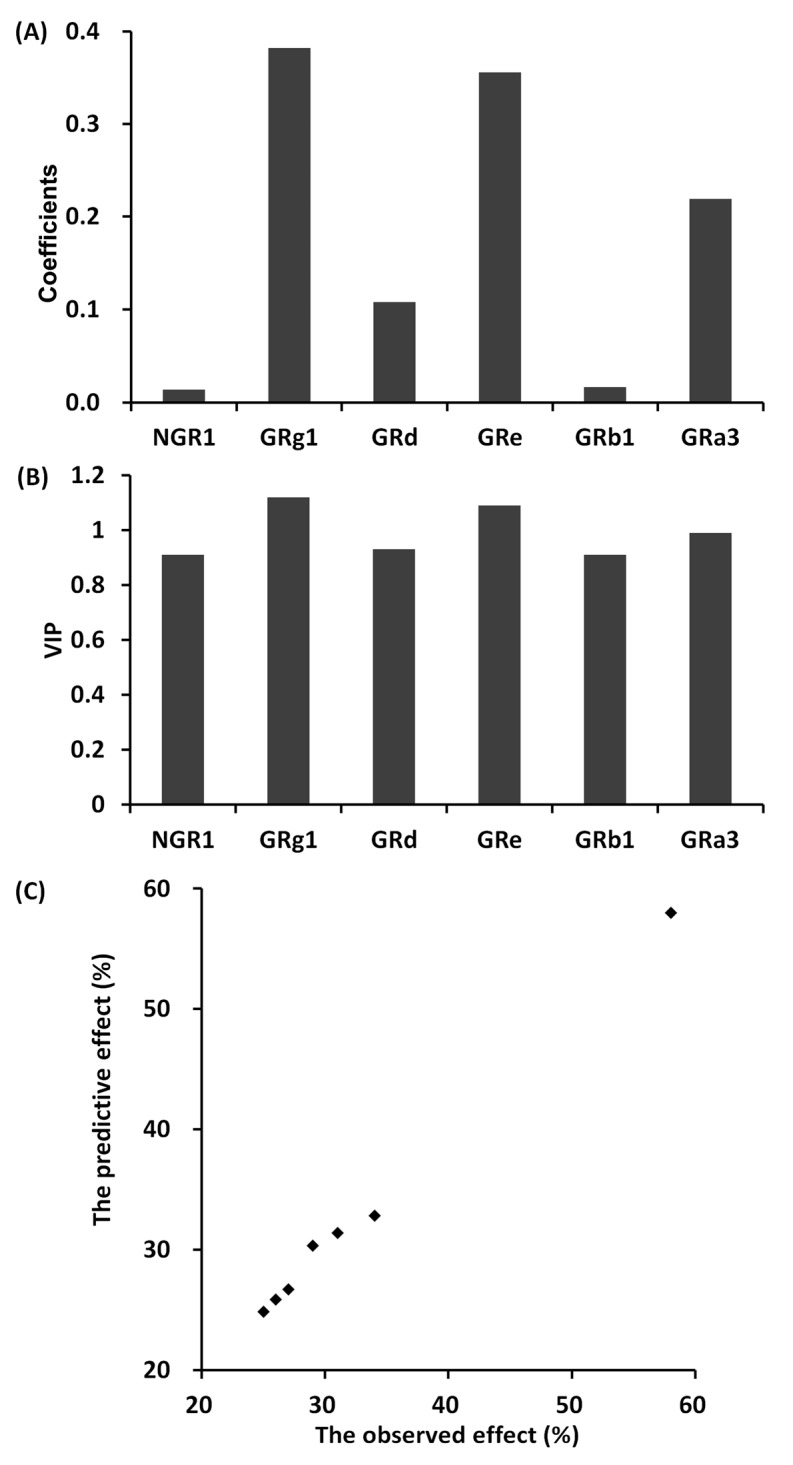
The partial least squares analysis between different individual saponin combinations and anti-platelet aggregation effect in vitro study in example 1. (**A**) The coefficients values of six saponins (GRb1, GRg1, GRd, NGR1, GRe, and GRa3); (**B**) The VIP values of six saponins (GRb1, GRg1, GRd, NGR1, GRe, and GRa3); (**C**) The diagram between the observed anti-platelet aggregation effect and the predictive anti-platelet aggregation effect.

**Figure 2:**
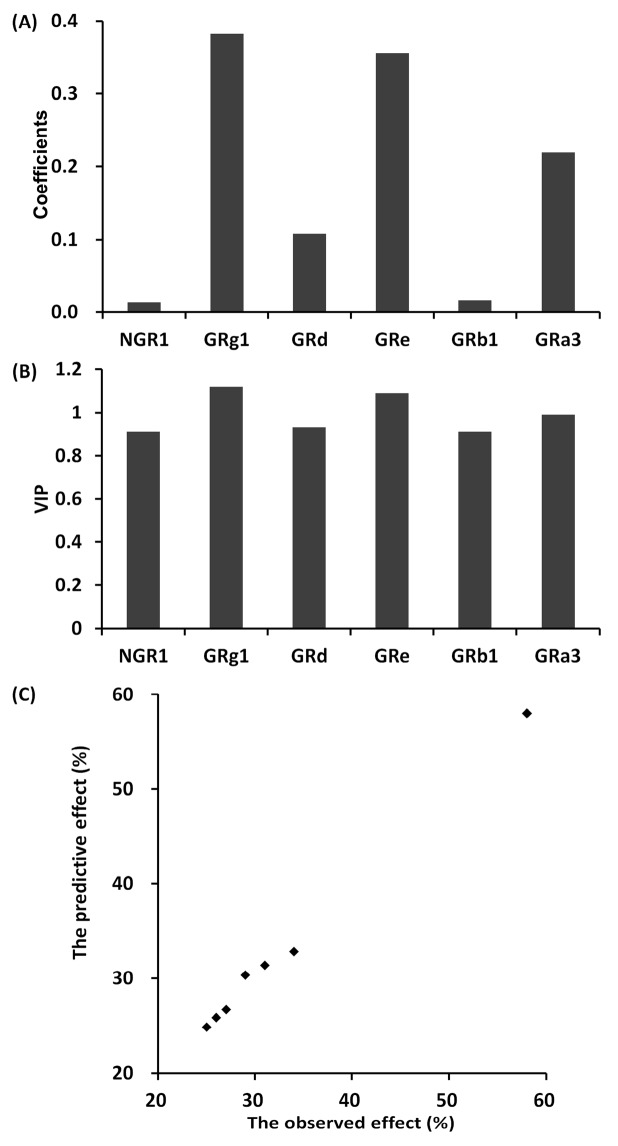
The partial least squares analysis between different individual saponin combinations and anti-platelet aggregation effect in vivo study in example 1. (**A**) The coefficients values of six saponins (GRb1, GRg1, GRd, NGR1, GRe, and GRa3); (**B**) The VIP values of six saponins (GRb1, GRg1, GRd, NGR1, GRe, and GRa3); (**C**) The diagram between the observed anti-platelet aggregation effect and the predictive anti-platelet aggregation effect.

The anti-platelet aggregation effects of seven different combinations of NGR_1_, GRg_1_, GRd, GRe, GRb_1_, and GRa_3_ on uniform design were shown in Table 2 and Table 3, respectively, for in vitro and in vivo. Based on these data sets, a partial least squares regression model was introduced to evaluate the relationship between six saponin content and the anti-platelet aggregation effect in different individual saponin combinations. In vitro, it produced a good partial least squares regression model with two principal components (R2X = 0.861, R2Y = 0.905, Q2Y = 0.883). The regression coefficients of NGR_1_, GRg_1_, GRe, GRb_1_, GRd, GRa_3_ on anti-platelet aggregations were 0.025, 0.371, 0.113, 0.346, 0.028, and 0.218, respectively (Figure 1A). Similarly, in vivo, it also produced a good partial least squares regression model with two principal components (R2X = 0.852, R2Y = 0.881, Q2Y = 0.864). The regression coefficients of NGR_1_, GRg_1_, GRe, GRb_1_, GRd, GRa_3_ on anti-platelet aggregations were 0.014, 0.382, 0.108, 0.356, 0.017, and 0.219, respectively (Figure 2A). The greater the coefficients value of an individual saponin, the more significant its effect on anti-platelet aggregations. The results of variable importance (VIP) values in vitro and in vivo also showed the contribution of each individual saponin to the anti-platelet aggregation effect (Figure 1B and Figure 2B), with the same trend as the regression coefficients. The larger the VIP value of an individual saponin, the greater its contribution to the anti-platelet aggregation effect. Importantly, the predicted anti-platelet aggregation curves were close to the observed data of dot marked by the partial least squares regression model (Figure 1C and Figure 2C). These results showed that the anti-platelet aggregation effect of different individual saponin combinations could be predicted accurately by this method.

### 3.2. Results of Fingerprint Studies (Example 2)

#### 3.2.1. The Fingerprint Studies of *Panax ginseng* Saponins Extract from Different Origins

Eighteen common peaks (P1–P18) of *panax ginseng* saponins extract from ten different origins (S1–S10) were identified from HPLC fingerprint study. P4 was identified to be ginsenoside Re (GRe) when compared with the reference standard of GRe. GRe is one of the main active components from our pharmacological studies, and the peak area of P4 was relatively higher with good separation resolution and symmetry, so P4 was chosen as the reference peak. For each component, the corresponding peak area was divided by the average area of P4 in ten *panax ginseng* saponins extract samples for non-dimensionalization (Table 4).

#### 3.2.2. The Effects of *Panax ginseng* Saponins Extract from Different Origins

The potential cardioprotective effects of *panax ginseng* saponins extract against ischemiareperfusion (IR) injury were estimated by determined the levels of cTnI, CK and LDH in cell medium (in vitro) and rat serum (in vivo). As shown in Table 5, compared with the sham control group, the cTnI, CK, and LDH values obtained in IR model group increased significantly, but they declined more or less after pretreatment by different batches of *panax ginseng* saponins extract from ten different origins (S1 to S10). The reversal degree among the ten batche saponins extracts was different. The S6 showed the strongest reversal at the cTnI value while S8 was the weakest. The S4 showed the strongest reversal at the CK and LDH values, while S9 was the weakest. Similarly, in assay of platelet aggregation inhibition, the platelet aggregation rate increased significantly in IR group as compared with the sham-control group, and then it decreased after the *panax ginseng* saponins extract was added. Among the ten batch saponins extracts, the platelet aggregation rate of S2 was the lowest (almost close to the sham control group), while the value of S1 was the highest.

#### 3.2.3. The Relevance between the Fingerprints and the Effects

**Figure 3:**
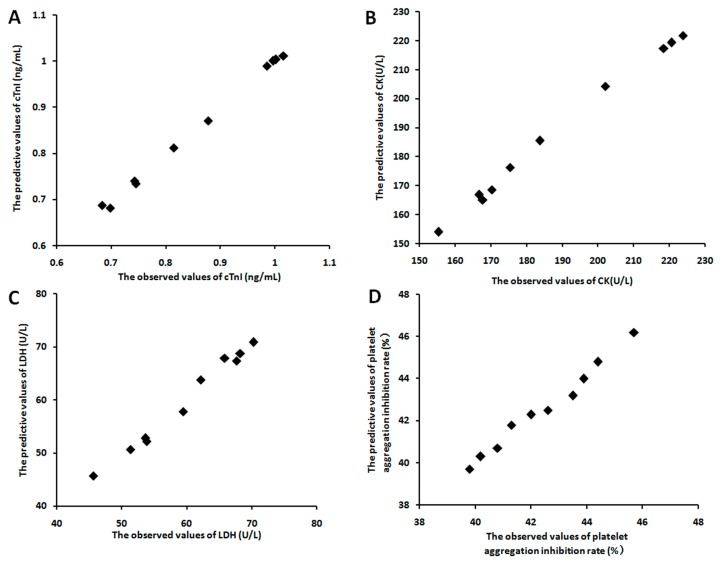
The diagram between the observed effects and the predictive effects in vitro studies in example 2. (**A**) cTnI; (**B**) CK; (**C**) LDH; (**D**) Platelet aggregation inhibition rate.

**Figure 4:**
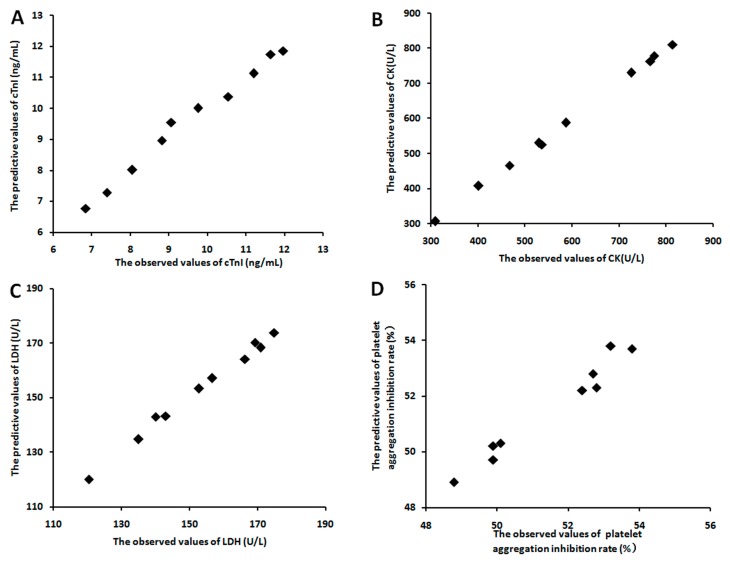
The diagram between the observed effects and the predictive effects in vivo studies in example 2. (**A**) cTnI; (**B**) CK; (**C**) LDH; (**D**) Platelet aggregation inhibition rate.

In order to evaluate the relationship between the four pharmacological markers and the eighteen components of *panax ginseng* saponins extracts, a partial least squares regression model was introduced. In vitro, it produced a good partial least squares regression model with two principal components (R2X = 0.83, R2Y = 0.87, Q2Y = 0.85), while a good partial least squares regression model with two principal components (R2X = 0.85, R2Y = 0.89, Q2Y = 0.83) was also produced in vivo. The regression coefficients of the eighteen components on the four pharmacological markers were shown in Table 6. The results of VIP values in vitro and in vivo also showed the contribution of each component to the four markers (Table 7). Most importantly, the predicted effect curves were close to the observed data of dot that was marked by the partial least squares regression model (Figure 3 and Figure 4). These results showed that the effects of different origins of *panax ginseng* saponins extract could be predicted accurately by this method.

## 4. Discussion

In this study, a partial least squares model was applied to investigate the relationship between the components content and pharmacological effect of traditional Chinese medicine. In example 1, firstly, the pharmacological effects of seven different individual saponin combinations were studied including six independent variables (six saponin doses) and one dependent variable (anti-platelet aggregation effect). The quantities of observations (*n* = 7) were relatively few for the six independent variables and one dependent variable. For this reason, the model fitting by the ordinary least squares regression will bias divergently. However, it was fitted well by the partial least squares model with a small number of observation. Secondly, the synergistic pharmacological effect of multiple components existed besides the individual effect of each component in traditional Chinese medicine. The ordinary multiple regression was not able to analyze the interactions among multiple components. But, the partial least squares model was able to analyze not only the correlations between the individual component and the pharmacological effect, but also the synergistic effect of multiple components. The predictive pharmacological effect of the different individual saponin combinations by the partial least squares model were close to the observed value, indicating the strong predictive function of the model. Furthermore, this method can also guide the optimization of the extraction process for traditional Chinese medicine by increasing the contents of components with high contribution to effect as much as possible. As this example, the contributions of GRg1, GRe, and GRa3 to anti-platelet aggregation effect of different saponin combinations were relatively larger according to the VIP diagrams (Figure 1B and Figure 2B). Thus, GRg1, GRe, and GRa3 had high positive correlation to the pharmacological effect, indicating the strong interpretation function of the model.

Before the multivariate statistics analysis in example 1, the anti-platelet aggregation effect between *panax ginseng* saponins extract and individual saponin combination (the dose of each saponin in individual saponin combination was equal to that of each saponin in *panax ginseng* saponins extract) were compared. The anti-platelet aggregation effect of the individual saponin combination was amount to 92% (in vitro) and 90% (in vivo) of the *panax ginseng* saponins extract, suggesting that the six saponins of NGR1, GRg1, GRd, GRe, GRb1, and GRa3 were the material basis of *panax ginseng* saponins extract for the antithrombotic effect. The total contents of the six saponins reached 86% in *panax ginseng* saponins extract. So, the investigations were designed to analyze the relationship between thecontentof multiple components and pharmacological effect by changing the individual saponin content in different individual saponin combinations. Therefore, there were two key preconditions in the application of this method. Firstly, the content of multiple the components in traditional Chinese medicine should be known, and the selected components should be the main material basis in herbal extract. Secondly, the selected components should have a major contribution to the pharmacological effect of herbal medicine. The partial least squares regression analysis could be applied to the multiple components content-effect analysis in traditional Chinese medicine if the two preconditions could be satisfied simultaneously.

The first attempt (example 1) was lack of the information of real conditions in traditional Chinese medicine. So we tried to apply the partial least squares method based on real combinations in example 2, to investigate the relationship between the fingerprint dataset and pharmacological effects of *panax ginseng* saponins extract. Firstly, the HPLC fingerprints of ten different batches of *panax ginseng* saponins extract from different origins were studied, and eighteen common peaks, regarded as eighteen independent variables, were identified in our study. Then, the protective effect son cardiovascular system of *panax ginseng* saponins extract were studied, and the cTnI, CK, LDH values and anti-platelet aggregation rate, regarded as four dependent variables, were selected as pharmacological indicators. Finally, a 18 × 10 X-matrix (fingerprints) and a 4 × 10 Y-matrix (effects) were formed from the raw data. This method can be used to accurately qualify the content-effect relationship of each component and predict the anti-myocardial ischemia-reperfusion injury directly by fingerprint datasets.

There are three important advantages of partial least squares analysis for fingerprints dataset of traditional Chinese medicines. First, more effective monomers of traditional Chinese medicine could be found, such as natural drugs. The relative contribution and the potential impact of each component on pharmacological effects would be analyzed by partial least squares method. After that, the monomers with high contributions could be separated, extracted, enriched, and purified, and then the pharmacological effects of these monomers were separately investigated to verify the partial least squares results. It would be an effective method to select active natural components from a large number of traditional Chinese medicines for new drug development. Second, the optimization of the extraction process for traditional Chinese medicines could be guided by our method. The amounts of active components in the traditional Chinese medicine would be selectively increased by improve the extraction method. Third, our method could provide useful information for clinical dosing regimens. Due to the pharmacological efficacy of traditional Chinese medicine, it can be speculated by our method according to the fingerprint datasets, that the dose regimens and the dose formulations of traditional Chinese medicine could be adjusted. For the modernization of traditional Chinese medicine, the study on the content-effect relationship have always been a key technology bottleneck, which was mainly due to the absence of appropriate methodology [29].Thus, the partial least squares analysis method effectively associated a large number of fingerprint spectrums with pharmacological data to form a more comprehensive research system to clarify multi-components content-effect relationships, which would be a great impetus for the research of herbal medicine.

## 5. Conclusions

In this manuscript, a multivariate regression statistic strategy was introduced to investigate the relationship between the content of multiple components and the pharmacological effects of panaxginseng saponins extract based on a partial least squares model. Both for the saponin combinations in example 1 and for the fingerprint spectrums in example 2, the content-effect correlation was fitted well by the partial least squares regression equations. The predicted effect curve was close to the observed data of dot marked on the partial least squares regression model. This study demonstrated that the multivariate regression statistic strategy could assess the contribution of each component to the efficacy of herbal medicine and, in turn, predict the potential efficacy of herbal medicine according to its components compositions. This would have important application value in the research and development of traditional Chinese medicine.

## Figures and Tables

**Table 1:** The contents of six saponins in *panax ginseng* saponins extract.

No. (%)	NGR_1_	GRg_1_	GRd	GRe	GRb_1_	GRa_3_
**1**	35.34	26.29	8.70	8.27	4.26	3.21
**2**	34.92	26.60	8.50	7.25	4.81	4.10
**3**	35.80	26.49	8.95	8.32	4.89	3.50
**4**	34.45	26.23	8.86	7.94	4.08	4.10
**5**	34.37	26.19	8.81	7.89	4.99	4.20
**MEAN**	34.98	26.36	8.76	7.93	4.61	3.82
**SD**	0.66	0.20	0.20	0.44	0.41	0.32

**Table 2:** The uniform design and the observed anti-platelet aggregation effect of seven different individual saponin combinations in vitro study in example 1.

No. (µg/mL)	NGR_1_	GRg_1_	GRd	GRe	GRb_1_	GRa_3_	Observed Effect (%)
**1**	0	5	0.5	40	4	4	30
**2**	1	40	2	20	0	1	33
**3**	2	2.5	8	10	8	0	26
**4**	4	20	0	5	1	8	28
**5**	8	0	1	2.5	16	2	24
**6**	16	10	4	0	2	0.5	25
**7**	32	80	16	80	32	16	59

**Table 3:** The uniform design and the observed anti-platelet aggregation effect of seven different individual saponin combinations in vivo study in example 1.

No. (mg/kg)	NGR_1_	GRg_1_	GRd	GRe	GRb_1_	GRa_3_	Observed Effect (%)
**1**	0	0.5	0.075	4	0.4	0.6	31
**2**	0.1	4	0.3	2	0	0.15	34
**3**	0.2	0.25	1.2	1	0.8	0	27
**4**	0.4	2	0	0.5	0.1	1.2	29
**5**	0.8	0	0.15	0.25	1.6	0.3	25
**6**	1.6	1	0.6	0	0.2	0.075	26
**7**	3.2	8	2.4	8	3.2	2.4	58

**Table 4:** Dimensionless data of peak areas of eighteen identified components (P1–P18) in ten different batches of *panax ginseng* saponins extracts from different origins (S1–S10) in example 2.

Batch	P1	P2	P3	P4	P5	P6	P7	P8	P9	P10	P11	P12	P13	P14	P15	P16	P17	P18
**S1**	0.543	0.430	0.580	0.894	0.577	0.183	0.520	0.598	0.646	0.308	0.349	0.255	0.473	0.239	0.177	0.583	0.485	0.671
**S2**	1.545	0.743	0.244	1.880	0.831	1.145	0.808	0.554	0.615	0.680	0.609	0.427	0.259	1.229	0.224	1.958	1.252	1.090
**S3**	0.923	0.844	0.703	1.090	1.185	1.831	0.420	2.504	0.983	0.415	0.531	0.662	0.223	0.271	1.095	0.649	1.063	0.525
**S4**	2.613	4.052	0.166	2.030	1.248	0.323	0.940	1.764	1.539	0.615	0.743	1.498	0.861	0.947	1.423	0.968	2.745	1.581
**S5**	0.148	0.081	0.820	0.290	0.362	0.285	0.173	0.546	0.239	2.479	0.212	0.284	0.115	0.821	0.453	0.802	0.414	0.198
**S6**	1.912	1.591	0.345	2.570	1.534	1.341	0.738	0.642	0.864	0.755	1.190	1.237	0.226	0.543	0.738	0.337	2.632	1.617
**S7**	1.485	1.672	0.664	1.340	0.780	0.938	0.501	0.436	1.030	0.976	0.683	0.104	0.299	2.816	1.167	0.939	1.203	0.929
**S8**	0.275	0.195	0.330	0.580	0.239	0.534	0.166	0.722	0.240	0.142	0.128	1.794	0.112	0.446	0.189	0.694	0.815	0.210
**S9**	0.374	0.314	0.779	0.460	0.211	0.621	0.167	0.755	0.229	0.387	0.119	0.579	0.109	0.606	0.500	0.325	0.580	0.391
**S10**	1.002	1.693	0.439	1.520	1.277	1.328	0.876	0.941	0.901	0.593	0.584	0.210	1.313	0.238	1.059	0.138	0.950	0.720

**Table 5:** The effects of ten different batches of *panax ginseng* saponins extracts (S1–S10) in vitro and in vivo study in example 2.

Groups	In Vitro Study				In Vivo Study			
	E1 (ng/mL)	E2 (U/L)	E3 (U/L)	E4 (%)	E1 (ng/mL)	E2 (U/L)	E3 (U/L)	E4 (%)
Sham	0.265	121.5	24.7	35.6	3.32	221.6	95.1	45.8
IR	1.217	267.8	90.4	53.2	13.75	986.7	183.4	57.9
IR + S1	0.997	218.4	67.6	45.7	11.97	766.9	169.2	54.1
IR + S2	0.743	170.4	53.8	39.8	9.06	536.3	143	48.8
IR + S3	0.815	175.5	59.4	42	9.75	587.1	152.8	52.4
IR + S4	0.699	155.4	45.6	41.3	7.39	308.7	120.5	50.1
IR + S5	0.986	202.1	65.8	43.5	10.54	726.5	166.3	52.8
IR + S6	0.684	166.8	51.3	40.2	6.84	401.3	135.1	49.9
IR + S7	0.746	167.8	53.6	40.8	8.05	467	140.1	49.9
IR + S8	1.016	220.5	68.2	44.4	11.64	775.1	170.9	53.2
IR + S9	1.002	223.7	70.2	43.9	11.2	814.4	174.9	53.8
IR + S10	0.878	183.8	62.1	42.6	8.81	529.3	156.7	52.7

Notes: IR, ischemia reperfusion; E1, cTnI; E2, CK; E3, LDH; E4, Platelet aggregation rates.

**Table 6:** The coefficients values of the eighteen components (P1–P18) of ten different batches of *panax ginseng* saponins extracts in vitro and in vivo study in example 2.

Type	Effect	P1	P2	P3	P4	P5	P6	P7	P8	P9	P10	P11	P12	P13	P14	P15	P16	P17	P18
In Vitro Study	E1	0.27	0.39	0.13	0.04	0.13	0.32	0.15	0.37	0.33	0.28	0.33	0.36	0.24	0.24	0.23	0.26	0.06	0.38
	E2	0.12	0.27	0.04	0.10	0.18	0.02	0.05	0.24	0.20	0.27	0.26	0.18	0.37	0.02	0.16	0.05	0.33	0.34
	E3	0.18	0.03	0.04	0.01	0.15	0.05	0.25	0.32	0.34	0.27	0.29	0.38	0.24	0.2	0.22	0.23	0.35	0.16
	E4	0.35	0.12	0.26	0.32	0.29	0.12	0.29	0.34	0.38	0.33	0.33	0.19	0.28	0.18	0.03	0.19	0.38	0.3
In Vivo Study	E1	0.26	0.37	0.16	0.03	0.17	0.33	0.17	0.39	0.34	0.21	0.33	0.38	0.21	0.23	0.28	0.29	0.05	0.39
	E2	0.13	0.27	0.02	0.11	0.17	0.04	0.04	0.22	0.19	0.25	0.26	0.2	0.36	0.04	0.18	0.04	0.37	0.36
	E3	0.19	0.04	0.03	0.02	0.11	0.06	0.24	0.33	0.30	0.31	0.36	0.31	0.22	0.16	0.21	0.25	0.31	0.21
	E4	0.33	0.18	0.23	0.30	0.25	0.13	0.28	0.37	0.35	0.29	0.30	0.22	0.19	0.19	0.05	0.19	0.37	0.29

Notes: E1, cTnI; E2, CK; E3, LDH; E4, Platelet aggregation rates.

**Table 7:** The VIP values of the eighteen components (P1–P18) of ten different batches of *panax ginseng* saponins extracts in vitro and in vivo study in example 2.

Type	Effect	P1	P2	P3	P4	P5	P6	P7	P8	P9	P10	P11	P12	P13	P14	P15	P16	P17	P18
In Vitro Study	E1	1.08	1.08	1.00	1.18	1.11	1.18	1.11	0.92	0.99	0.97	1.06	1.01	1.17	1.05	0.98	0.96	1.06	1.13
	E2	0.91	1.00	0.91	1.04	1.15	1.09	1.13	1.03	1.12	0.94	1.19	1.09	1.06	1.16	1.09	1.12	1.09	0.91
	E3	1.08	0.92	0.99	1.17	1.01	1.01	0.93	1.05	0.99	0.93	1.15	0.92	1.1	1.1	0.99	0.94	1.13	0.98
	E4	1.17	0.97	0.97	1.09	0.91	1.16	1.14	1.06	1.19	0.98	1.15	0.92	1.04	1.08	1.05	1.1	1.19	1.07
In Vivo Study	E1	1.03	1.05	1.01	1.12	1.06	1.05	1.10	0.91	0.97	0.95	1.02	1.02	1.15	1.06	0.98	0.99	1.01	1.12
	E2	0.91	1.02	0.92	0.99	1.13	1.10	1.12	1.01	1.14	0.93	1.15	1.07	1.15	1.18	1.07	1.13	1.08	0.93
	E3	1.08	0.95	0.96	1.14	0.99	1.00	0.96	1.04	0.98	0.94	1.17	0.93	1.08	1.07	1.02	0.97	1.15	1
	E4	1.18	0.99	0.97	1.10	0.93	1.14	1.14	1.07	1.18	0.97	1.11	0.93	1.02	1.06	1.04	1.08	1.13	1.02

Notes: E1, cTnI; E2, CK; E3, LDH; E4, Platelet aggregation rates.
